# No increase in corticospinal excitability during motor simulation provides a platform to explore the neurophysiology of aphantasia

**DOI:** 10.1093/braincomms/fcae084

**Published:** 2024-03-21

**Authors:** Maaike Esselaar, Paul S Holmes, Matthew W Scott, David J Wright

**Affiliations:** Department of Life Sciences, Manchester Metropolitan University, Manchester M1 5GD, UK; Department of Sport and Exercise Sciences, Manchester Metropolitan University, Manchester M1 7EL, UK; School of Kinesiology, University of British Columbia, Vancouver, Canada V6T 1Z1; Department of Psychology, University of British Columbia, Kelowna, Canada V1V 1V7; Department of Psychology, Manchester Metropolitan University, Manchester M15 6GX, UK

## Abstract

This scientific commentary refers to ‘Explicit and implicit motor simulations are impaired in individuals with aphantasia’, by Dupont  *et al*. (https://doi.org/10.1093/braincomms/fcae072) in *Brain Communications*

**This scientific commentary refers to ‘Explicit and implicit motor simulations are impaired in individuals with aphantasia’, by Dupont  *et al*. (** https://doi.org/10.1093/braincomms/fcae072 **) in *Brain Communications***

The study by Dupont *et al*.^1^ published in *Brain Communications* used single-pulse transcranial magnetic stimulation (TMS) to measure corticospinal excitability during visual imagery, kinaesthetic imagery and action observation of a pinch movement. Participants self-reported either typical imagery abilities or aphantasia (reduced or absent imagery abilities).^2^ The study represents the first example of a TMS experimental paradigm being applied for aphantasia research. Dupont *et al*.^1^ report the well-established effect that corticospinal excitability is facilitated by kinaesthetic imagery and action observation^3^ in participants with typical imagery generation abilities. In contrast, however, these facilitation effects were not found in the participants reporting aphantasia, where corticospinal excitability was not facilitated by either the explicit (kinaesthetic imagery) or implicit (action observation) simulation of movement. These findings, therefore, provide new evidence that the experience of aphantasia may be underpinned by an inability of the brain to simulate actions, rather than a failure in volitional effort or metacognition. This is a novel finding that contributes to the growing body of literature exploring the mechanisms underpinning aphantasia^2^ and points towards a possible altered neural signature for this individual difference. In this commentary, we highlight methodological issues that may warrant further investigation, before focusing on future research opportunities for the neurophysiology of aphantasia.

Dupont *et al*.’s^1^ team has an established profile in TMS research and motor imagery processes^3^ and used accepted and rigorous TMS methods to conduct the study. Appropriate procedures were used to determine the optimal scalp position and resting motor threshold, to ensure consistent TMS coil placement throughout the study, and to confirm no significant differences in resting pre-stimulation electromyography activity that could potentially skew the data. One potential methodological issue, however, relates to the timing at which the TMS pulses were delivered. Single-pulse TMS to the motor cortex provides a marker of corticospinal excitability that is time-locked to the point of stimulation delivery.^3^ In the two imagery conditions, TMS was delivered at 2000 ms into a 3000-ms imagined isometric finger–thumb pinch contraction. The aphantasic participants would, therefore, have had to not only generate a kinaesthetic image but also maintain it for 2000 ms for facilitation in corticospinal excitability to be detected. Some aphantasic individuals report being able to generate vague and dim visual imagery, yet they may struggle to maintain it for this duration, and so it is plausible that an increase in corticospinal excitability may have been identified had the stimulation been delivered earlier during the imagined kinaesthetic contraction. In addition, during the action observation condition, TMS was delivered at 1000 ms after the observed contact between the index finger and thumb. Facilitation of corticospinal excitability is greatest during action observation when delivered at the point of maximal muscle contraction in the observed muscles^4^ (i.e. at least 1000 ms earlier than the stimulation delivery by Dupont *et al*.^1^). It would, therefore, be useful for future research to vary the stimulation timings to confirm that the null effects reported in the aphantasia group do indeed represent a deficit in image generation abilities, rather than stimulation timing decisions or image maintenance mechanisms. Further, the researchers delivered only 16 stimulations per condition, and, although justified, there is good evidence to recommend that 24–30 stimulations are preferable to ensure a more reliable estimate of corticospinal excitability.^5^ Although these concerns do not necessarily cast doubt on the findings reported by Dupont *et al*.,^1^ future replication attempts may wish to vary the stimulation timings and increase the number of TMS trials per condition to give further support for the finding of the current study.

Dupont *et al*.’s^1^ use of TMS to explore aphantasia provides informative and novel findings; however, TMS only indexes cortico-cortical and cortico-spinal activity. Given the comments above concerning image generation and image maintenance within visual and kinaesthetic modalities, we suggest it would be worthwhile for future research to also explore motor simulation abilities in aphantasic participants using alternative techniques such as functional MRI to establish if and where any simulation impairment may occur. For example, if image generation deficits underpin aphantasia, then disruption to networks in posterior occipital cortex may be observed. Maintenance and transformation issues may arise elsewhere in parietal and temporal networks. Similarly, we suggest it may also be fruitful to consider the implicit and explicit imagery procedures in the context of the visual processing systems as recent evidence from area V1 indicates implicit visual imagery, but not explicit visual imagery, to be intact in aphantasia.^6^

Establishing the effects of visual imagery, kinaesthetic imagery and action observation on corticospinal excitability was a useful first step for motor simulation research in aphantasia, and the findings reported by Dupont *et al*.^1^ provide potentially mechanistic evidence for a motor simulation deficit in this population. While aphantasia is often described as a deficit in visual imagery abilities, Dawes *et al*.^2^ have indicated that aphantasia could be more heterogenous and encompass simulation deficits across multiple and interacting modalities (i.e. kinaesthetic, auditory, olfactory, tactile, etc.). Dawes *et al*.^2^ estimated that 24% of aphantasics experience a total multi-sensory absence of imagery ability and that 30% experience aphantasia in only the visual modality, with the remaining 46% experiencing intact imagery abilities across one or more different modalities. Figure 1a of Dupont *et al*.^1^ indicates that participants had relatively low visual and kinaesthetic imagery abilities, without differentiating between visual only and multi-sensory aphantasics. It would be interesting to establish whether corticospinal excitability would be facilitated during motor simulation in certain aphantasia sub-types, such as those with reduced visual imagery ability but intact kinaesthetic imagery ability, or whether the effects identified by Dupont *et al*.^1^ replicate across all aphantasia sub-types. Future researchers replicating the Dupont *et al*. study may, therefore, benefit from including a multi-sensory imagery ability questionnaire and comparing the effect on corticospinal excitability of motor simulation in different aphantasia sub-types.

Dupont *et al*.’s^1^ novel findings provide a platform for future research exploring the effects of motor simulation across the spectrum of imagery abilities and imagery modalities. Individual differences in visual imagery ability characteristics have been categorized on a spectrum, with aphantasia at one extreme.^7^ At the other, individuals who report visual imagery that is as clear and vivid as real vision are said to experience hyperphantasia.^7^ An interesting future study would be to replicate Dupont *et al*.’s^1^ study with the inclusion of individuals who self-report the experience of hyperphantasia. It is conceivable that delivering TMS during motor simulation conditions to those who experience hyperphantasia may facilitate corticospinal excitability to a greater extent than those with typical visual imagery abilities, and such an effect could form a useful biomarker of hyperphantasia when combined with other techniques.

Another avenue for future research could be to explore the effects of combined action observation and motor imagery (AOMI) where participants watch movements displayed on video while imagining simultaneously the kinaesthetic sensations associated with movement execution.^8^ There is evidence that, in those with typical imagery abilities, AOMI facilitates corticospinal excitability to a greater extent than action observation and to a comparable extent to motor imagery.^9^ In AOMI conditions, the need to generate visual imagery is reduced as visual information is provided by the video content, which serves as a visual guide with which the participant can synchronize their kinaesthetic imagery.^8^ If aphantasia is characterized predominantly by reduced visual imagery abilities,^2^ it is possible that the video element of AOMI may provide a ‘scaffold’ to allow aphantasics to generate the kinaesthetic imagery of movement. A logical next step would, therefore, be to establish whether individuals with aphantasia can self-report an ability to engage in AOMI and whether corticospinal excitability is facilitated in aphantasic participants during AOMI conditions. As ∼1 in 25 individuals (4%) experience aphantasia,^10^ such a finding would support the use of AOMI as a more accessible intervention format than independent motor imagery techniques used widely to support motor performance and learning. An interesting further study would therefore be to replicate the study by Dupont *et al*.,^1^ with the inclusion of an AOMI condition.

In conclusion, the findings of the study by Dupont *et al*.^1^ advance the scientific understanding of aphantasia by providing evidence that aphantasia is not a difference in metacognition but may be underpinned by neurophysiological deficit. With some methodological issues to consider, the novel findings reported in this study contribute significantly to the aphantasia literature. Dupont *et al*.^1^ should be commended for providing the platform for future research in motor simulation across the visual imagery ability spectrum. Three interesting avenues for future investigation would be to explore these effects using TMS and other neurophysiological measures: (i) across both multi-sensory as well as visual only aphantasics, (ii) in hyperphantasics as well as aphantasics, and (iii) during AOMI conditions as well as during independent imagery and observation.

## References

[1] Dupont W, Papaxanthis C, Madden-Lombardi C, Lebon F. Explicit and implicit motor simulations are impaired in individuals with aphantasia. Brain Commun. 2024;6:fcae072. 38515440 10.1093/braincomms/fcae072 PMC10957132

[2] Dawes AJ, Keogh R, Pearson J. Multisensory subtypes of aphantasia: Mental imagery as supramodal perception in reverse. Neurosci Res. 2023. 10.1016/j.neures.2023.11.009 38029861

[3] Grosprêtre S, Ruffino C, Lebon F. Motor imagery and cortico-spinal excitability: A review. Eur J Sport Sci. 2016;16(3):317–324. 25830411 10.1080/17461391.2015.1024756

[4] Gangitano M, Mottaghy FM, Pascual-Leone A. Phase-specific modulation of cortical motor output during movement observation. NeuroReport. 2001;12(7):1489–1492. 11388435 10.1097/00001756-200105250-00038

[5] Cuypers K, Thijs H, Meesen RLJ. Optimization of the transcranial magnetic stimulation protocol by defining a reliable estimate for corticospinal excitability. PLoS One. 2014;9(1):e86380. 24475111 10.1371/journal.pone.0086380 PMC3901672

[6] Cabbai G, Racey C, Simner J, Dance C, Ward J, Forster S. Sensory representations in primary visual cortex are not sufficient for subjective imagery. *bioRxiv*. 2024. 10.1101/2024.01.10.574972 39419033

[7] Zeman A, Milton F, Della Sala S, et al Phantasia—The psychological significance of lifelong visual imagery vividness extremes. Cortex. 2020;130:426–440. 32446532 10.1016/j.cortex.2020.04.003

[8] Eaves DL, Hodges NJ, Buckingham G, Buccino G, Vogt S. Enhancing motor imagery practice using synchronous action observation. Psychol Res. 2022. 10.1007/s00426-022-01768-7. PMC11315722 36574019

[9] Chye S, Chembila Valappil A, Wright DJ, et al The effects of combined action observation and motor imagery on corticospinal excitability and movement outcomes: Two meta-analyses. Neurosci Biobehav Rev. 2022;143:104911. 36349570 10.1016/j.neubiorev.2022.104911

[10] Dance CJ, Ipser A, Simner J. The prevalence of aphantasia (imagery weakness) in the general population. Conscious Cogn. 2022;97:103243. 34872033 10.1016/j.concog.2021.103243

